# Severe Airway Epithelial Injury, Aberrant Repair and Bronchiolitis Obliterans Develops after Diacetyl Instillation in Rats

**DOI:** 10.1371/journal.pone.0017644

**Published:** 2011-03-25

**Authors:** Scott M. Palmer, Gordon P. Flake, Fran L. Kelly, Helen L. Zhang, Julia L. Nugent, Patrick J. Kirby, Julie F. Foley, William M. Gwinn, Dan L. Morgan

**Affiliations:** 1 Division of Pulmonary and Critical Care Medicine - Duke University Medical Center, Durham, North Carolina, United States of America; 2 National Institute of Environmental Health Sciences, Research Triangle Park, North Carolina, United States of America; Centre de Recherche Public de la Santé (CRP-Santé), Luxembourg

## Abstract

**Background:**

Bronchiolitis obliterans (BO) is a fibrotic lung disease that occurs in a variety of clinical settings, including toxin exposures, autoimmunity and lung or bone marrow transplant. Despite its increasing clinical importance, little is known regarding the underlying disease mechanisms due to a lack of adequate small animal BO models. Recent epidemiological studies have implicated exposure to diacetyl (DA), a volatile component of artificial butter flavoring, as a cause of BO in otherwise healthy factory workers. Our overall hypothesis is that DA induces severe epithelial injury and aberrant repair that leads to the development of BO. Therefore, the objectives of this study were 1) to determine if DA, delivered by intratracheal instillation (ITI), would lead to the development of BO in rats and 2) to characterize epithelial regeneration and matrix repair after ITI of DA.

**Methods and Main Results:**

Male Sprague-Dawley rats were treated with a single dose of DA (125 mg/kg) or sterile water (vehicle control) by ITI. Instilled DA resulted in airway specific injury, followed by rapid epithelial regeneration, and extensive intraluminal airway fibrosis characteristic of BO. Increased airway resistance and lung fluid neutrophilia occurred with the development of BO, similar to human disease. Despite rapid epithelial regeneration after DA treatment, expression of the normal phenotypic markers, Clara cell secretory protein and acetylated tubulin, were diminished. In contrast, expression of the matrix component Tenascin C was significantly increased, particularly evident within the BO lesions.

**Conclusions:**

We have established that ITI of DA results in BO, creating a novel chemical-induced animal model that replicates histological, biological and physiological features of the human disease. Furthermore, we demonstrate that dysregulated epithelial repair and excessive matrix Tenacin C deposition occur in BO, providing new insights into potential disease mechanisms and therapeutic targets.

## Introduction

Bronchiolitis obliterans (BO) is a fibrotic lung disease characterized by narrowing and obliteration of the small airways.[1] BO is most often recognized to occur in the setting of lung or bone marrow transplantation, but has also been described in the setting of occupational exposure to reactive volatile chemicals.[2] Once BO occurs patients develop irreversible airflow obstruction that can progress to respiratory failure, depending on the clinical context. BO is distinguished from cryptogenic organizing pneumonia which involves intraalveolar and intraluminal airway fibrosis, often occurs after infection, and frequently improves with treatment. Due to the difficulty in confirming histological BO in the setting of lung or bone marrow transplantation, the term bronchiolitis obliterans syndrome (BOS) has been developed to identify patients with presumed BO based on declining lung function in the absence of other etiologies.[3], [4] Despite the clinical importance of BO, little is known about its pathogenesis, and there are no effective treatments to reverse the airway fibrosis. The lack of intervention and treatment strategies for BO can be attributed in part to the limited availability of animal models in which to study disease development. An overall goal of our research is to develop a relevant rodent model of BO in which to further investigate disease mechanisms.

Diacetyl (DA), the major volatile component of artificial butter flavoring, has been associated with the development of airflow obstruction and BO in workers in the microwave popcorn, [5], [6] [7] flavoring[8], and diacetyl manufacturing industries[9]. Consistent with these clinical reports, acute DA inhalation in rats was shown to cause inflammation and necrosis of the epithelium of the nasal cavity and upper airways [10]. Similarly, we found that subacute or repeated inhalation of DA caused inflammation and necrosis of the nasal passages of mice [11]. Although pulmonary symptoms predominate in exposed workers, the nasal cavity was the site of the most severe tissue damage in DA-exposed rats and mice [5] [10], [11]. Interestingly, BO-like lesions did not develop in either of these rodent inhalation models. Because rodents, unlike humans, are obligate nose breathers, we believe that inhalation leads to extensive absorption and reaction of DA in the nasal cavity preventing penetration to the distal airways. In fact, inhaled dosimetry and computational modeling studies performed with DA vapor in the rat suggest that nasal and tracheal injury in the rat might be predictive of intrapulmonary injury in mouth breathing humans.[12]

Given these previous results, we hypothesized that bypassing the nasal cavity by intratracheal instillation (ITI) of DA would lead to the development of BO in rats. Although a non-physiological route of exposure, ITI of chemicals, such as bleomycin, has proven to be a very useful strategy for investigating mechanisms of toxicity to the respiratory tract leading to fibrosis [13]. In order to test this hypothesis we assessed histological, biological and physiological measures and demonstrate the utility of instilled DA as a relevant BO model. Furthermore, we hypothesized that ITI of DA induces severe epithelial injury and aberrant repair that leads to the development of BO. In order to test this hypothesis, we then characterized serial chronological changes in airway epithelial cells and matrix components from the time of DA exposure to the development of BO. Using this approach, we have established that aberrant epithelial and matrix repair occur in the setting of DA-induced BO.

## Materials and Methods

### Ethics statement

All of the scientific data was performed with high technical, scientific, and ethical standards. All use of vertebrate animals is approved by the intramural National Institute of Health NIEHS Animal Care and Use Committee (ACUC), NIEHS Assurance number A4149-1. NIH guidelines are followed to ensure that numbers of animals and animal care is appropriate, and that the required measures are taken to limit animal discomfort, distress, pain and injury. Methods of euthanasia and reason for selection are consistent with the AVMA Guidelines on Euthanasia. Veterinary care is provided by highly trained and experienced staff.

### Chemicals

DA (2,3-butanedione), CAS#431-03-8, was purchased from Fluka Chemical Company (Milwaukee, WI) as a single lot number 1131562. The purity was ≥99.0%. Bradford reagent (CN 23208) was purchased from Sigma-Aldrich (St. Louis, MO). LDH Reagent kit (CN-L7572) was purchased from Pointe Scientific (Canton, MI).

### Animals

Male, Sprague-Dawley rats (295–305 g body weight) were obtained from Charles River (Raleigh, NC). During a 5–10 day acclimation, rats were weighed and randomized to treatment groups. Animals were housed individually and provided with NIH-31 chow and water *ad libitum*. Animal rooms were maintained with a light-dark cycle of 12 h (light from 0700 to 1900 h). Body weights were recorded on the day prior to treatment and 1, 3, and 7 days after treatment. Subgroups of animals were weighed and euthanized (Nembutal, 60 mg/kg ip and pneumothorax) at 1, 3, and 7 days after treatment for endpoint analysis as described in the sections below.

### Intratracheal Instillation

A mean body weight determined on study day 1 was used in dose calculation. DA (188 mg/ml) was prepared in a ventilated hood on the morning of dosing using sterile distilled water as the vehicle. On study day 0, animals were lightly anesthetized (isofluorane) and then intratracheally-instilled with DA (125 mg/kg), or vehicle as a control. The volume instilled (200 µl) was the same for all animals.

### Pulmonary Function Assessment

Pulmonary function was measured invasively in a subset of rats 7 days after treatment with DA (n = 8) or vehicle (n = 4) using the FlexiVent mechanical ventilator and data acquisition system (SCIREQ, Montreal, PQ, Canada). Rats were anesthetized (Nembutal, 60 mg/kg ip), a tracheal cannula inserted, and then paralyzed with pancuronium bromide (0.82 mg/kg, ip). A differential pressure transducer was connected to the tracheal cannula and ventilation was maintained at a rate of 90 breaths/minute, a tidal volume of ∼8 ml/kg and a positive end expiratory pressure of ∼3 cm of water. For each rat, three total lung capacity (TLC) maneuvers were performed to prevent atelectasis and to maximize airway and alveolar recruitment prior to the initiation of lung mechanics measurements which then cycled a total of 9 times per animal. Values for total resistance (R), dynamic compliance (C) and static compliance (Cst) obtained after each cycle were averaged for each animal and these values were then averaged within each experimental group.

### Necropsy

At 1, 3, and 7 days after treatment, animals (n = 6–9/group) were euthanized (Nembutal, 60 mg/kg ip, pneumothorax) and the lungs removed *en bloc*. The right bronchus was ligated and the right lower (caudal) and accessory lobes were removed and placed in cold RNAlater (Ambion, Austin, TX), then stored at 4°C until RNA extraction. The left lung was then inflated with OCT (50% OCT, 50% 0.9% saline). Sections of the left lung were placed in a cryomold containing OCT, placed on dry ice and frozen for immunofluorescence microscopy. Remaining segments of left lung were fixed in 10% neutral-buffered formalin. The remaining right upper (cranial) and middle lung lobes were inflated with 10% neutral-buffered formalin, the bronchus ligated and the lobes immersed in formalin. After 24 hours in formalin, the right lobes and segments of left lung were transferred to 70% ethanol and refrigerated until processed for light microscopy.

### Bronchoalveolar lavage fluid (BALF) cell counts

Subgroups of rats (n = 6) were euthanized 1, 3, and 7 days after treatment with DA or vehicle and the lungs lavaged 3x with 10 ml cold Ca^++^,Mg^++^-free Hanks balanced salts solution (BSS). The BALF samples were centrifuged 500× g for 10 minutes at 4°C. The cell pellets from the 3 BALF samples were combined and the total number of BAL cells/rat determined electronically (Coulter ZB, Coulter Electronics, Inc., Marietta, GA). Cell differentials were determined from manual cell counts of stained cytospin preparations.

### Histopathology

After fixation in 10% neutral-buffered formalin followed by 70% ethanol, sections of the right and left lungs were routinely processed, embedded in paraffin, serial-sectioned at 5 µm and stained with hematoxylin and eosin (H&E) and Masson's trichrome. The magnification of images was estimated by multiplying the objective of the lens (10–40x) by the magnification of the photomultiplier tube (10x) to provide the total magnification. To evaluate cell proliferation, subgroups of animals (n = 12) were pulse-dosed with 50 mg/kg (0.5 ml ip) bromodeoxyuridine (BrdU) 4 hours prior to euthanasia. A section of duodenum was included in each cassette to confirm systemic delivery of BrdU to the animal.

The presence of BO was defined by airway obstruction due to intraluminal polypoid fibrosis or concentric fibrosis within the airway wall. To determine the extent of BO among DA treated and control animals, the incidence of bronchial and bronchiolar fibrotic lesions was assessed at days 3 (9 rodents/group) and 7 (9 rodents in DA, 6 in control group). Bronchial and bronchiolar fibrosis was graded in a semi-quantitative manner by an experienced lung pathologist. The designation of intraluminal or intramural bronchial and bronchiolar fibrosis was based upon the predominant location of the lesion (e.g. primarily within the lumen or primarily within the wall of the airway) and scored as follows: 1–5 lesions  = 1+, 6–10 lesions  = 2+, 11–15 lesions  = 3+, and >15 lesions  = 4+. The score reflects only bronchial or bronchiolar fibrosis and not fibrosis of the alveolar ducts, alveolar spaces or alveolar walls. Although all lobes of the right and left lung have been examined in different experiments and have contained BO lesions, the fibrosis score in the current study was based solely upon representative sections of the left lung and upper and middle lobes of the right lung since the remainder of lung tissue was used for fluorescent stains or RNA analysis.

### Immunofluorescence

Sections of lung were frozen (left) or formalin fixed (right and left) at necropsy. Frozen tissue was preserved at −80°C until cryosectioned at 6 µm. Sections were stored at −80°C until stained. Formalin fixed paraffin embedded tissue was serial-sectioned at 5 µm and the slides stored at room temperature until stained.

Primary Antibodies: Rabbit anti-rat Clara cell secretory protein (CCSP) antibody (1∶20,000; gift from Barry Stripp), mouse IgG2b anti-acetylated tubulin (AcT) antibody (1∶24,000, Sigma, St Louis, MO), mouse IgG2a anti-smooth muscle actin (SMA) antibody (1∶500, Sigma, St Louis, MO), mouse IgG1 anti-BrdU antibody (1∶400,San Jose, CA), and rabbit anti-human Tenascin-C antibody (1∶200, Santa Cruz Biotechnology, Santa Cruz, CA). Secondary antibodies: Alexa Fluor 594 Donkey anti-Rabbit IgG, Alexa Fluor 488 Goat anti-mouse IgG2b, Alexa Fluor 488 Goat anti-mouse IgG2a, and AlexaFluor 488/594 Goat anti-mouse IgG1 (1∶500, Invitrogen, Carlsbad, CA). Sections were blocked with 5% BSA in phosphate-buffered saline (PBS) for non-specific antigen reactivity following citrate buffer retrieval. Primary and secondary antibodies were diluted in blocking solution and incubated overnight at 4°C or room temperature for 90 minutes, respectively. Slides were extensively washed in PBS and cover-slipped with DAPI (Sigma, St Louis, MO) in Fluoromont G mounting media (Southern Biotech, Birmingham, AL). Images were obtained using an Olympus Provis AX70 microscope (Center Valley, PA) equipped with a digital camera and processed using Image-Pro Plus (Media Cybernetics, Silver Spring, MD).

### Whole lung transcript expression

Each analysis at the various time points represents a minimum of 6 separate animals in treated vs. control groups. For transcriptional analysis, the caudal and accessory lobes of the right lung were placed into RNAlater™. RNA isolation was performed using the RNAqueous-4PCR kit (Ambion, Austin, TX) and quantified by a spectrophotometer (260/280) (NanoDrop products, Wilmington, DE). Quality was assessed by the Bio-Rad Experion bioanalyzer (Bio-Rad, Hercules, CA). RNA was reverse transcribed into cDNA using the high capacity reverse transcription kit (ABI, Foster City, CA). Transcripts for genes of interest (Clara Cell Secretory protein, surfactant protein C, collagen 1alpha-1, and Tenascin C) were assessed using 40 ng of cDNA in each assay of Taqman realtime PCR using validated Taqman probe and primer combinations (ABI, Foster City, CA); each assay was performed in triplicate. Cycle conditions were: 95°C for 10 minutes, and 40 cycles of 95°C for 15 seconds and 60°C for 1 minute. RT-PCR for target transcripts and endogenous transcripts (β actin) were performed in multi-plex using Fam-labeled target probes, and VIC-labeled endogenous probes. Ct values were determined using an ABI 7500 RealTime PCR System (ABI, Foster City, CA) with SDS software version 1.3.1. Fold change was determined by comparing DA dCt values to same day water dCt values, and calculated using the 2^-ΔΔCt^ method.[14]

### Statistical analysis

Changes in BAL fluid cell counts were determined by 1-way ANOVA and Tukey's multiple comparison test. For analysis of bronchial and bronchiolar fibrosis score and pulmonary function parameters (R, C and Cst) unpaired Student's t tests were used. For analysis of transcript data, non parametric t-tests were used to compare differences among means in two independent groups. All of these analyses were performed using PrismGraphPad 5. P-values of less than 0.05 were considered statistically significant.

## Results

### DA treated animals develop histopathological changes characteristic of BO

All of the DA treated rodents, but none of the controls, developed bronchial and bronchiolar fibrotic lesions. These foci of airway fibrosis were present in major bronchi as well as in bronchial branches and bronchioles. When all lobes were examined across multiple experiments, the lesions were more numerous in the left lung and in the right lower and accessory lobes than in the right upper and middle lobes. As shown in Table 1, using a semi-quantitative scoring system that reflects the extent of bronchial and bronchiolar lesions, BO developed consistently among all the day 7 DA-treated rodents but not in the vehicle-treated animals and increased in severity from day 3 to day 7 after DA treatment (bronchial and bronchiolar fibrosis score at day 7 in DA-treated rodents vs. vehicle controls, 3.0±0.33 vs. 0±0, respectively, p value  = 0.004).

**Table 1:** Effect of Diacetyl on Airway Fibrosis.

Time after Treatment	Treatment Group	Incidence of Lesions^a^	Fibrosis Lesions/rat^b^	Bronchial and Bronchiolar Fibrosis Score^c^	p-value^d^
Day 3	diacetyl	7/9	0–9	1.0±0.2	0.02
	vehicle	0/6	0	0±0	
Day 7	diacetyl	9/9	6–23	3.0±0.3	0.004
	vehicle	0/6	0	0±0	

a Number of rats with lesions/number evaluated.

b Range of lesions.

c Mean ± SE.

d diacetyl vs. vehicle control, Wilcoxon sign rank test.

**Figure 1:**
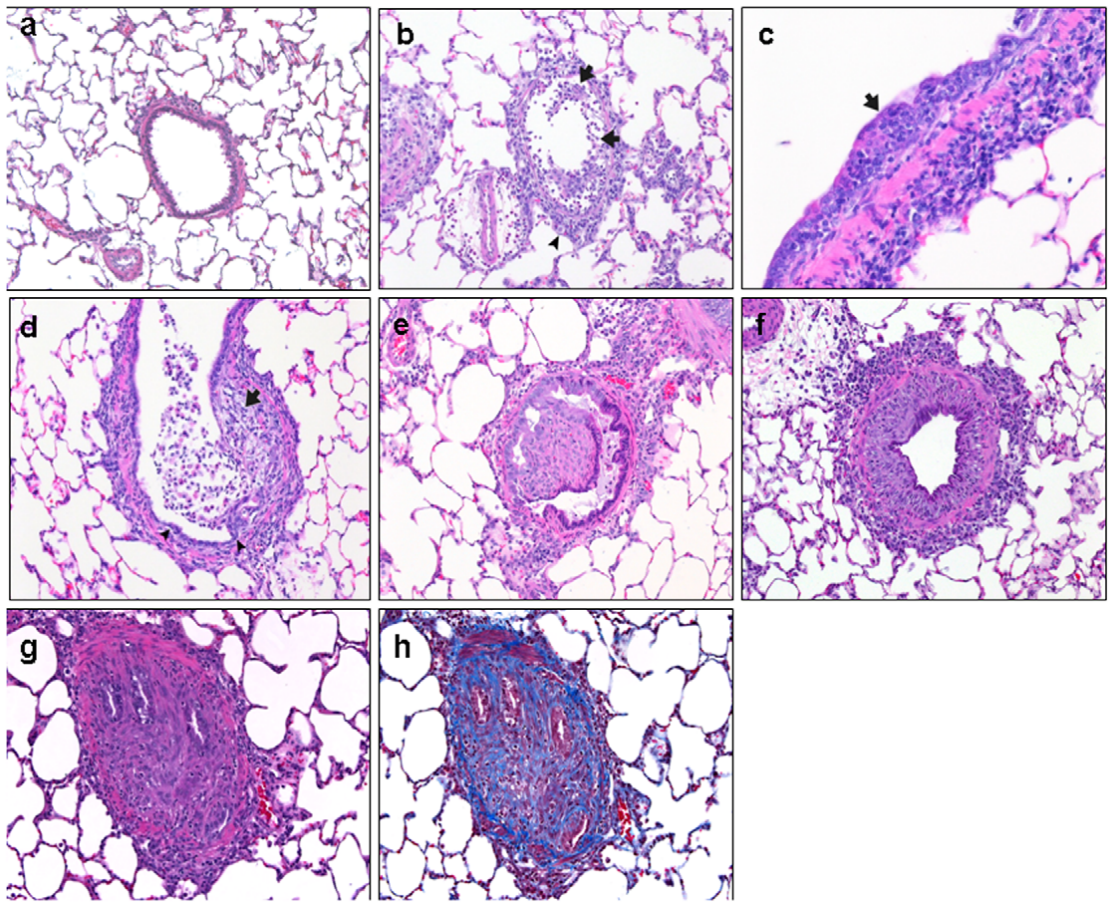
Progression of epithelial injury to bronchiolitis obliterans after diacetyl instillation. a, Bronchiole of a control rodent 7 days post instillation, showing normal airway epithelium and structure (H&E, 200x). b, Bronchiole of a diacetyl- (DA-) treated rodent 1 day after instillation showing epithelial sloughing (arrows) and peribronchiolar inflammation (arrowhead) (H&E,100x). c, Bronchus of DA-treated rodent 3 days after instillation showing regenerating epithelium with mild hyperplasia (arrow), (H&E, 200x). d, Bronchiole of DA-treated rodent 3 days after instillation showing fibrosis (arrow) in area of epithelial erosion, regenerating epithelium in bottom portion of bronchiole (arrowheads), and mixed intraluminal inflammation (H&E, 100x). e, BO in a bronchiole characterized by polypoid intraluminal fibrosis and peribronchiolar inflammation (H&E, 100x). f, Bronchiole with concentric intramural fibrosis, typical of constrictive BO (H&E, 100x). g,h, Advanced BO lesion with nearly complete airway obstruction. g, Extensive intraluminal occlusion with only a few epithelial remnants (H&E, 200x). h, Extensive intraluminal collagen and fibrosis (blue) (Masson's Trichrome, 200x).

Figure 1 shows the progression of epithelial injury leading to BO. In contrast to a vehicle-treated control airway (Figure 1a), ITI of DA resulted in severe necrosis and sloughing of the bronchial and bronchiolar epithelium by day 1 (Figure 1b). Mixed populations of inflammatory cells, including histiocytes, lymphocytes, eosinophils, and neutrophils were present in and around bronchi with epithelial necrosis. By day 3, evidence of regenerating epithelium was present with moderate epithelial hyperplasia in some airways and frequently a surrounding adventitial wreath of mononuclear cells composed of histiocytes and lymphocytes (Figure 1c). In addition, by day 3, areas of polypoid intraluminal airway fibrosis were evident in association with regions of epithelial erosion (Figure 1d). At this stage, the intraluminal fibrosis had a loose, myxoid appearance. By day 7, more extensive airway fibrosis accompanied by a mixed inflammatory infiltrate was evident, resulting in partial obstruction of airways either by intraluminal polypoid growths (Figure 1e) or by intramural (constrictive or circumferential) fibrosis (Figure 1f). A few airways exhibited virtually complete obstruction of lumens by dense fibrous tissue (Figure1g) with a prominent collagenous component evident on the Masson Trichrome stain (Figure 1h).

### Increased neutrophilia occurs in the lung fluid of DA-treated animals

The total number of BAL cells increased significantly 1 and 3 days after ITI DA, but returned to levels comparable to controls by day 7 (Table 2). BAL neutrophils; however, were significantly elevated at days 1, 3, and 7 in DA-treated rodents as compared to controls. The numbers of alveolar macrophages were significantly greater than controls only at day 1.

**Table 2:** Neutrophil Influx into the Lung Fluid of Diacetyl-treated Rats.

	Treatment Group^a^	Day 1^b^	Day 3 b	Day 7 b
Total Cells	vehicle	4.91±1.72	4.23±1.95	5.84±0.89
	diacetyl	9.42±2.59*	8.71±2.36*	5.14±1.30
Macrophages	vehicle	3.81±1.42	4.16±2.03	5.84±0.89
	diacetyl	6.31±2.16*	5.28±1.68	4.03±2.10
Neutrophils	vehicle	0.89±1.00	0.06±0.08	0.00±0.00
	diacetyl	3.10±0.24*	3.07±1.12*	1.11±1.46*

a 5–9 animals/group.

b Mean cell number (x10^6^) ± SD.

*Significantly different (p<0.05) from respective controls.

### DA-treated animals develop abnormal lung function consistent with severe small airways disease

**Figure 2:**
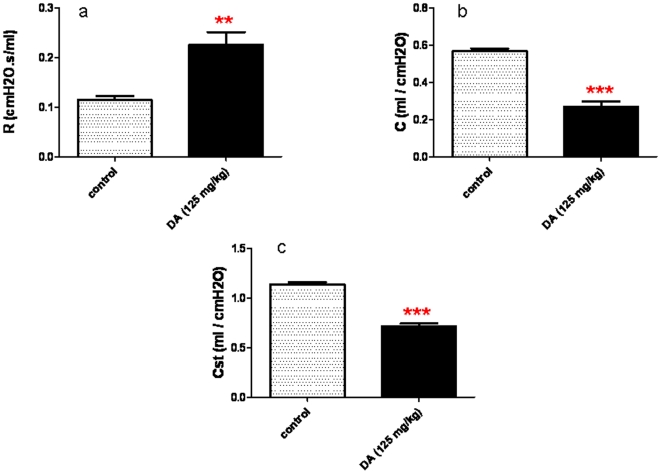
Altered Lung Function in Diacetyl-treated Rats. Pulmonary function was evaluated 7 days after treatment with diacetyl (DA) or water (control). a, Airway resistance (R), was significantly increased (**p<0.01) in DA-treated rats, relative to controls. b,c, Both dynamic (C) and static (Cst) compliance were significantly decreased (***p<0.001) in DA-treated rats, relative to controls.

DA-treated animals as compared to controls demonstrated significant changes in pulmonary function at Day 7. Airway resistance (R) was significantly increased (*p*<0.01) in DA-treated rodents relative to controls (Figure 2a). As shown in Figures 2b and 2c, both dynamic (*p*<0.001) and static compliance (*p*<0.001) were significantly decreased in DA-treated rodents relative to controls.

### DA treated animals develop an epithelial specific pattern of injury with fibrosis

**Figure 3:**
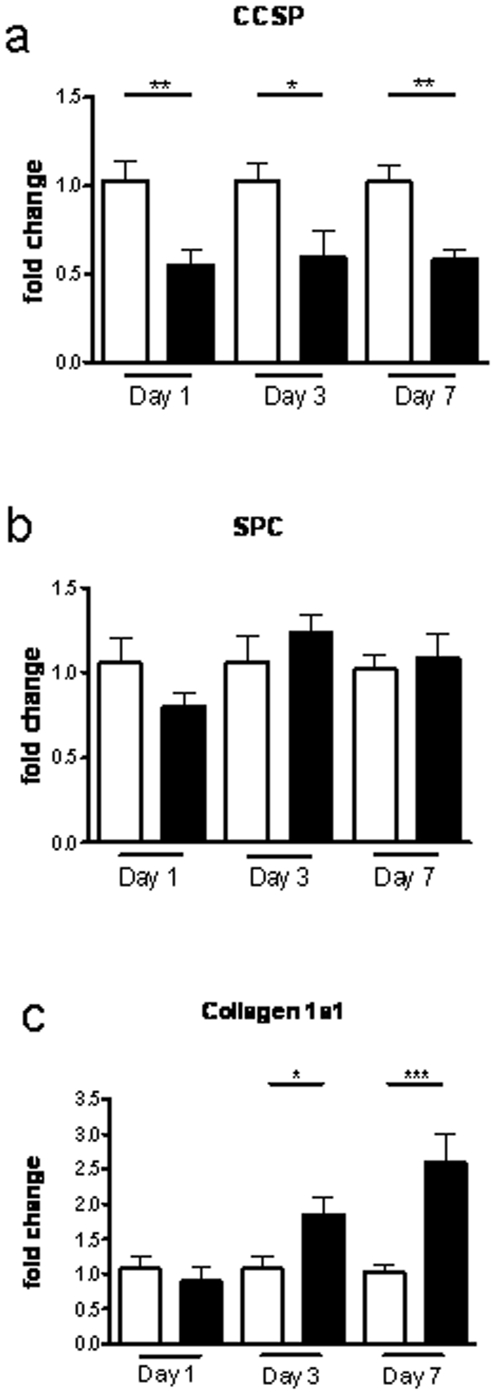
Diacetyl exposure results in early epithelial loss of CCSP transcript followed by collagen deposition. Whole lung transcript analysis using real-time PCR mRNA levels from rodents treated with water or diacetyl (DA). At each time-point (day 1, 3, and 7) DA-treated rodents were all compared to their appropriate water treated counterparts. a, CCSP transcript is significantly down-regulated at days 1,3, and 7. b, SPC transcript is minimally affected 1 day post DA treatment, and recovers to expression levels similar to water control at days 3 and 7. c, Collagen 1α-1 transcript is significantly up-regulated at days 3 and 7 post DA treatment, consistent with BO histology.

Whole lung transcript analysis, as shown in Figure 3a, demonstrates a significant depletion of the epithelial transcript CCSP, a marker present exclusively in the non-ciliated Clara cells, showing a 1.8-fold down regulation (p = 0.005), at day 1. Furthermore, depletion of CCSP transcripts persisted through day 3 (1.7 fold down-regulation, p = 0.02) and day 7 (1.7 fold down regulated, p = 0.003) after DA exposure. In contrast, levels of surfactant protein-C (SPC) present in alveolar cells (Figure 3b), demonstrated only minimal down-regulation (1.2 fold down-regulated, p = 0.18) at day 1, and transcript levels returned to control levels at days 3 and 7, thus confirming a pattern of injury specific to the airway epithelium and not the parenchymal tissue. Figure 3c shows collagen 1α-1 transcript was up-regulated 1.74-fold (p = 0.043) at day 3, with an even greater 2.6-fold increase at day 7 (p = 0.0007), as compared to controls, consistent with the observed airway fibrosis in Figure 1e-h.

### DA induces rapid and severe loss of the normal airway epithelium

**Figure 4:**
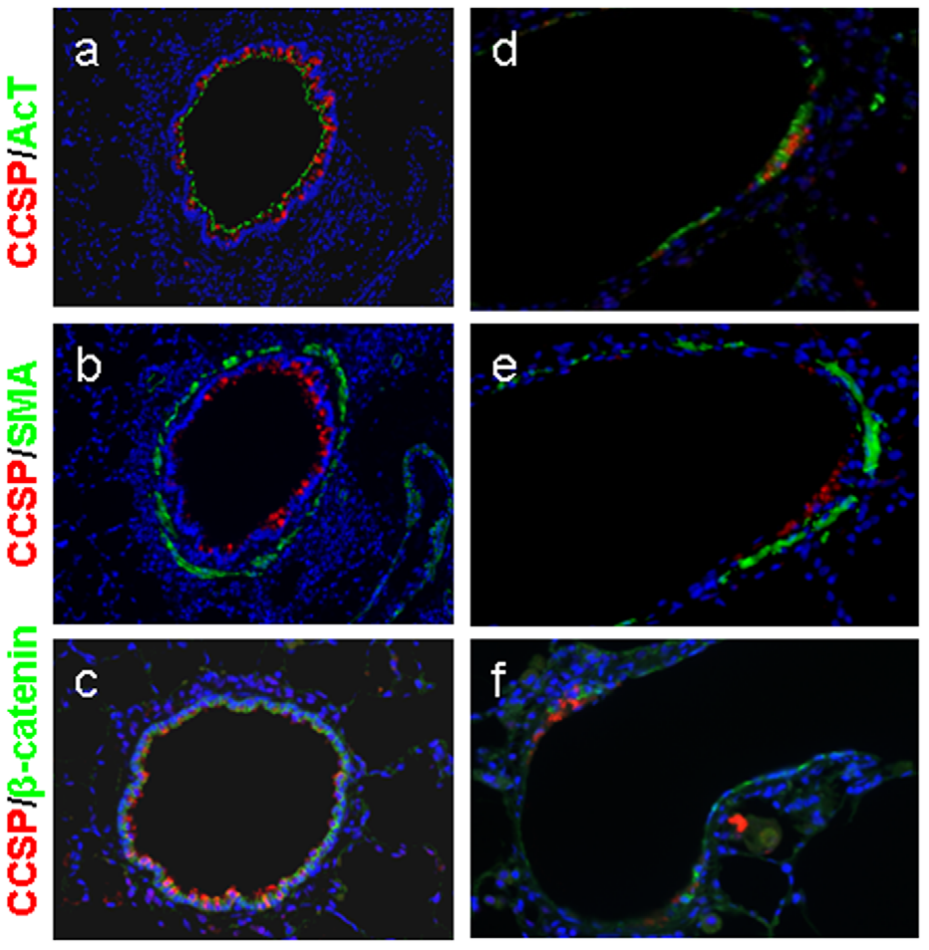
Severe epithelial injury with loss of normal epithelial structure occurs one day after Diacetyl treatment. a, d, Co-immunodetection of CCSP (red), and acetylated tubulin (AcT), (green). b, e, Co-immunostained for CCSP (red), and SMA (green). c, f, Co-immunostained for CCSP (red) and β-catenin (green). DAPI, a nuclear counter-stain, is shown in blue. Airways from control rodents (a,b,c) show baseline expression in controls of CCSP, AcT, SMA, and β-catenin (200x). CCSP and AcT (a) are expressed throughout a small airway, with a thin layer of SMA (b) surrounding the airway. The β-catenin staining shows membrane associated expression (c). Airways from DA-treated rodents one day following exposure (d, e, f) demonstrate severe depletion of epithelial protein expression of CCSP, AcT (d), and β-catenin (f), (400x). The β-catenin staining (f) also illustrates a flattened post injury cellular structure.

Immunohistochemical stains for CCSP, acetylated tubulin (AcT), smooth muscle actin (SMA), and β-catenin of control rodents show the normal distribution of airway epithelial cell types, normal columnar membrane expression of β-catenin, and minimal bronchiolar smooth muscle (Figure 4a–c). One day following ITI of DA, only small remnants of the normal epithelium remained with a few cells present expressing CCSP, AcT, or β-catenin (Figure 4d–f). Furthermore, the epithelial remnants in Figure 4f appear flattened and with suggestive intracellular, rather than junctional, staining of β-catenin (Figure 4f).

### Airway epithelium proliferates after DA treatment

**Figure 5:**
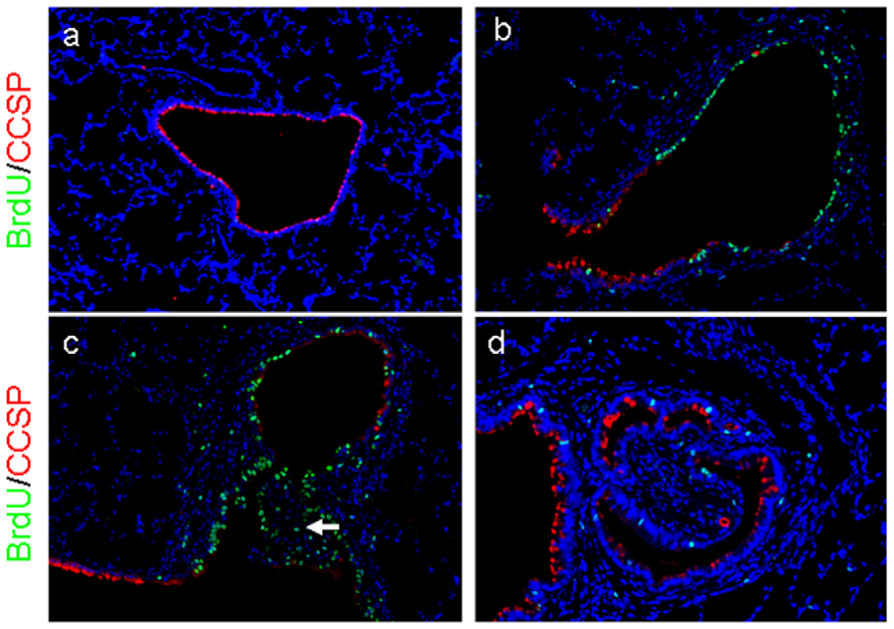
Regeneration of injured epithelium after diacetyl instillation. a, b, c, d, Co-immunodetection of CCSP (red) and BrdU (green), with DAPI, a nuclear counter-stain (blue). a, An airway from a control rodent at day 3 shows expected distribution of CCSP with no BrdU detection (100x). b, An early lesion from a rodent treated with diacetyl (DA) shows significant epithelial regeneration detected by BrdU by day 3 (200x). c, A moderate BO lesion seen at day 3 after DA exposure shows significant BrdU staining along the epithelium as well as within the fibrotic lesion (arrow) (200x). As seen in b and c, regions of regeneration do not co-localize with epithelial CCSP staining. d, In an advanced BO lesion at day 7, CCSP distribution remains abnormal and diminished as compared to normal, and regeneration is also diminished (200x).

BrdU staining demonstrated rapid regeneration of the bronchial and bronchiolar epithelium in DA-treated rodents. Although minimal BrdU staining was evident in both groups at day 1 (data not shown), by day 3 the airway epithelium of DA-treated rodents demonstrated extensive increased proliferation, not seen in the vehicle controls, (Figures 5a–c). Interestingly, evidence of increased BrdU activity was also evident within the regions of intraluminal polypoid fibrosis at day 3 (Figure 5c), but not present at day 7, in the setting of an advanced nearly obliterated airway (Figure 5d).

### Aberrant airway epithelial cell regeneration occurs in BO

**Figure 6:**
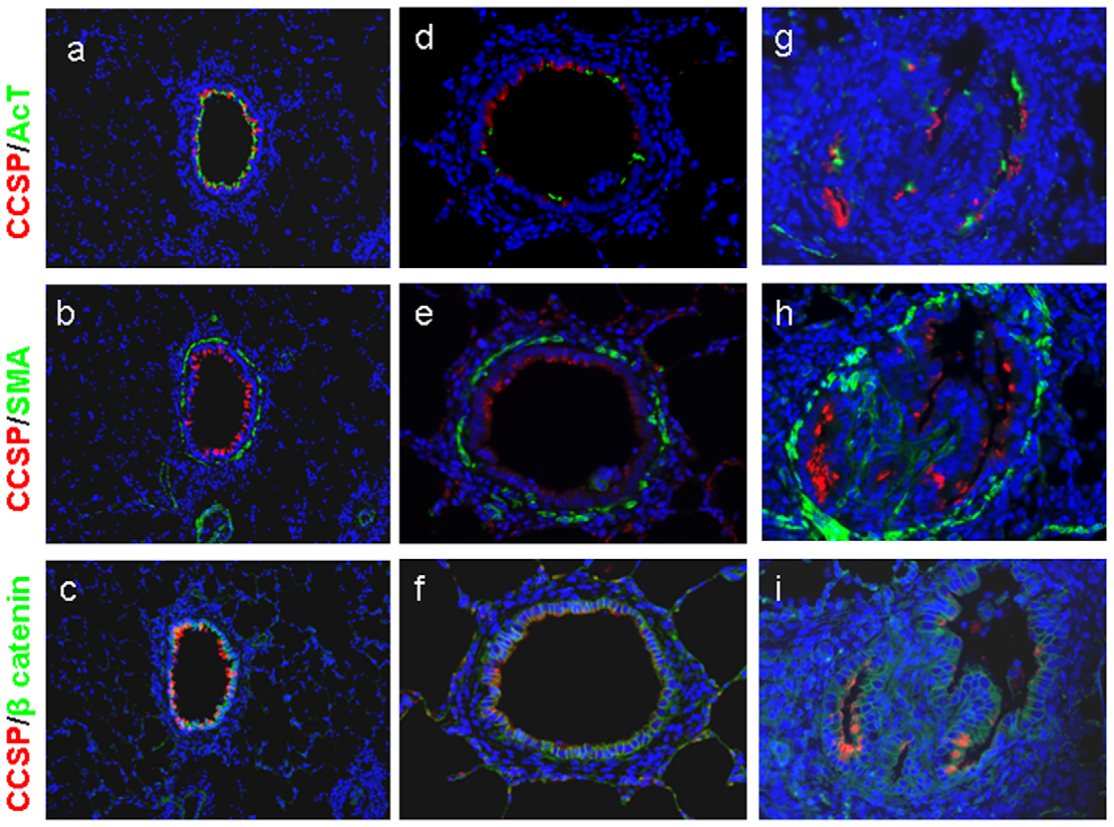
Injured epithelium regenerates, shows abnormal cellular composition, and progresses to BO. a, d, g, show co-immunodetection of CCSP (red) and acetylated tubulin (AcT) (green). b, e, h, are co-immunostained for CCSP (red), and SMA (green). c, f, i, are co-immunostained for CCSP (red) and β-catenin (green). DAPI, a nuclear counter-stain, is shown in blue. a, b, c, represent an airway from a control rodent (200x). d, e, f, represent an early lesion from a DA-treated rodent 3 days after instillation (200x). g, h, i represent an advanced lesion airway from a DA-treated rodent 7 days after instillation (400x). Three days after DA, the β-catenin expression appears normal, showing that epithelial repopulation has occurred (f), but CCSP and AcT expression is markedly diminished (d, e) demonstrating that the repopulated epithelium does not exhibit the normal airway cellular composition. Similar to day 3, the advanced day 7 BO lesion also shows a loss of CCSP and AcT expression; however, β-catenin expression is also irregular reflecting the distorted epithelium overlying the intraluminal polypoid lesions.

Immunohistochemical stains for CCSP, AcT, SMA, and β-catenin of control rodents show the normal distribution of airway epithelial cell types with a majority of cells expressing CCSP or AcT, normal junctional expression of β-catenin, and with minimal airway SMA (Figure 6a–c). In contrast, in a minimally affected airway of a representative early (day 3) lesion (Figure 6d–f), expression of both CCSP and AcT were diminished as compared to controls despite relatively normal appearing epithelial junctional β-catenin expression. Furthermore, in an advanced day 7 lesion with severely obliterated airway, β-catenin expression, although disorganized, was present covering regions of intraluminal fibrosis (Figure 6i). In contrast, there is almost no CCSP or AcT expression within the epithelium (Figure 6g,h). In addition, SMA labeling shows focal staining suggestive of myofibroblasts in areas of active intraluminal fibrosis, and bronchiolar SMA that appears more abundant and disorganized than in controls (Figure 6h).

### DA induced BO is characterized by excessive matrix deposition of Tenascin C

**Figure 7:**
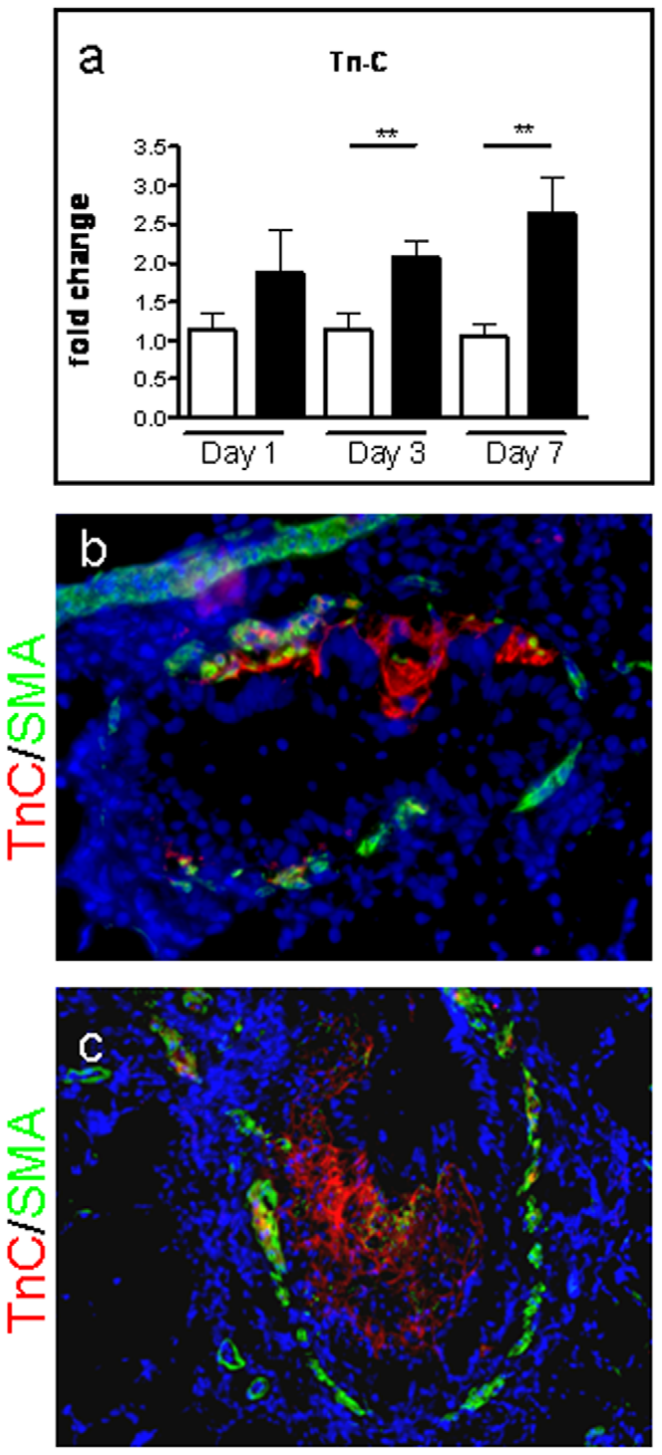
Tenascin-C transcript and protein is up-regulated after diacetyl treatment. Real-Time PCR showing mRNA levels derived from total whole lung RNA from rodents treated with water or diacetyl (DA). At each time-point (day 1, 3, and 7) DA-treated rodents were all compared to their appropriate control counterparts. a, Tn-C transcript is significantly up-regulated at days 3 and 7, following DA exposure. b, c, Images show co-immunodetection for Tn-C (red), and SMA (green). DAPI, a nuclear counter stain, is shown in blue. b, An early day 3 lesion of a medium-sized affected airway shows Tn-C expression within an area of active intraluminal fibrosis, while SMA is predominately detected in the wall of the airway (200x). A larger airway, c, representing an advanced day 7 lesion also shows Tn-C deposition throughout the fibrotic BO lesion (200x).

Transcript expression of the matrix component Tenascin C (Tn-C) was up-regulated at all three time points tested after DA exposure, and significantly so at days 3 (2.07-fold, p = 0.008) and 7 (2.64-fold, p = 0.018) as compared to controls (Figure 7a). Immunohistochemical staining confirmed intraluminal Tn-C protein expression in airways 3 and 7 days following DA exposure. Small airways with active BO demonstrated accumulation of Tn-C expression within early intraluminal polypoid lesions (Figure 7b), and diffusely throughout advanced BO lesions (Figure 7c).

## Discussion

We present a novel chemical-induced rodent model of BO using ITI administration of DA and demonstrate that this model replicates physiological, biological and pathological features of human BO. Within seven days after a single high-dose DA exposure, rats develop increased airway resistance, increased BAL neutrophilia, and airway fibrosis and histopathology characteristic of BO. In contrast to other rodent models used to study BO such as heterotopic tracheal transplantation, in our model airway fibrosis develops in the setting of a fully vascularized and aerated lung parenchyma[15]. This easily reproducible and clinically relevant model should advance our understanding of the pathogenesis and potential treatment of BO.

DA was selected to model the development of BO given the strong clinical and epidemiological associations between workplace exposure to DA and BO. In previous studies, rodents exposed to DA by inhalation failed to develop BO-like lesions, although extensive necrosis occurred in the nasal cavities of treated rats and mice [10], [11]. Because rodents are obligate nose breathers, we hypothesized that the extensive absorption and reaction of DA in the nasal cavity prevents toxic concentrations of DA from reaching the distal airways when delivered by inhalation, consistent with previous dosimetry studies in rats treated with DA vapor.[12] In addition, intratracheal administration of other chemical agents, such as nitric acid or gastric contents, has been associated with bronchiolar changes in rat and canine models.[16], [17] Therefore, in order to bypass the nasal cavity, DA was administered by ITI in the current study. Although this model is useful for investigating the pathogenesis of BO, given the nonphysiological route of administration, our results may have limited use for human risk assessment.

We have demonstrated that a single bolus intratracheal delivery of high-dose DA will cause BO in rodents. In contrast, human studies suggest that BO develops in factory workers exposed to DA or other volatile components of artificial butter flavorings as a result of constant or intermittent inhalational exposure to considerably lower concentrations of vapors. We are actively pursing additional studies in rodents to better characterize the long-term effects of inhaled DA at various concentrations as well as ITI of other related compounds present in the flavoring mix. These inhalation studies could provide a direct biological link between current workplace DA exposures and the development of human BO.

Several features of our model are particularly notable and closely parallel human BO. The hallmark lesion of BO, small airway fibrosis, was observed in all DA-treated animals. The presence of fibrosis was confirmed with increased trichrome staining in the lesions and significantly increased collagen transcripts within the lung. In addition, the physiological airflow measurements provide another strong biological link to human disease. A clinical diagnosis of BO is characterized by obstructive physiology with a reduced FEV_1_ (forced expiratory volume in 1 second). Although FEV_1_ cannot be measured in rats, measurements of increased airway resistance and decreased compliance are accepted indicators of airway obstruction in rodents.[18] In the setting of DA induced BO we observed significantly increased airway resistance and decreased compliance. Given the normal appearance of most of the lung parenchyma and airway specific pattern injury observed with DA exposure these results would suggest that most of the airways were affected and at least partially occluded in the DA treated animals. Since the small airways in normal lungs only make a minimal contribution to total airway resistance the significant changes observed in this study could only occur with very extensive small airway disease.

Furthermore, neutrophilic airway inflammation in this model also parallels that described in human BO. Neutrophilic inflammation has been reported in microwave popcorn factory workers with obstructive lung disease in association with the development of other indices of pulmonary inflammation [19]. Increased neutrophils in the lung fluid also have been extensively described in the setting of transplant related BO [20], [21]. Additional studies are needed to clarify the extent to which neutrophils participate in the development of inflammatory injury to the airway epithelium, degradation of the basement membrane, or fibroblast activation in the setting of DA-induced BO.

Although precise mechanisms that lead to the development of DA-induced BO are uncertain, several intriguing findings are apparent from our results. First, BO appears to result from an initial severe injury to the lower airway epithelium by this highly reactive chemical. DA is a strong electrophile and would be expected to readily react with thiols and amines on cellular proteins. Damage to membrane proteins by DA could compromise membrane integrity resulting in loss of the airway epithelium. In some early lesions we observed sloughing of intact epithelial cells, suggesting that DA may specifically affect epithelial attachment sites with or without necrosis of epithelial cells. In either case, loss of the overlying epithelium and disruption of the basement membrane could be prerequisites for airway fibrosis. For example, one plausible mechanism would involve severe epithelial injury leading to exposed fibrous lamina propria, allowing fibroblast precursors to migrate from the ulcerated airway wall into the lumen.

A very striking and novel observation is that despite rapid epithelial regeneration after DA injury the airway cellular composition remains abnormal. We demonstrated a marked reduction in protein expression of CCSP and AcT despite apparent epithelial regeneration. Furthermore we confirm depletion of CCSP transcript at all time points tested after DA exposure. These changes might simply reflect part of the normal process of epithelial repair and a failure of newly differentiated epithelium to express normal cellular markers. Alternatively, aberrant epithelium cellular composition might contribute directly to the development of intraluminal fibrosis, perhaps through an inability of the newly regenerated epithelium to limit fibroblast activity. Additional research is needed to determine if disruption of the airway epithelial layer is also integral to the pathogenesis of BO in DA-exposed workers or transplant recipients.

Given that a subset of CCSP expressing epithelial cells have been implicated to function as progenitor cells critical for normal repair of the lower airway epithelium, the sustained depletion of these cells, in particular, could contribute to disease pathogenesis [22]. A plausible hypothesis, consistent with our results, would be that the loss of Clara cells with their progenitor capacity contributes to aberrant local epithelial repair mechanisms that lead to the development of BO. Further studies are needed to determine if the loss of CCSP actively contributes to BO, or is simply a consequence of the disease development. In either case, these findings provide another strong parallel between our model and human BO, as a depletion of CCSP has been reported in the blood or lung fluid of lung or bone marrow transplant recipients with BO [23], [24].

Another novel observation of our study is the upregulation of Tn-C both at a transcript and protein level in BO. Tn-C is a matrix protein that has recently been identified to accumulate in several inflammatory and fibrotic lung conditions. For example, increased Tn-C has been reported in the airway walls of human asthmatic patients and in murine models of asthma [25], [26]. Furthermore, Tn-C deficient mice have reduced airway hyperreactivity, cell recruitment and inflammatory cytokines after ovalbumin asthma challenge, suggesting a role for this matrix protein in the promotion of lung inflammation [27]. Finally, up-regulation of this matrix protein was recently reported in a murine model of aberrant epithelial repair [28]. In these studies, naphthalene exposure was used as a model of productive or successful epithelial repair and contrasted with persistent (non-productive) Clara cell injury occurring as a result of ganciclovir administration to transgenic mice (CCtk) expressing herpes simplex virus thymidine kinase (HSV-tk). In the case of naphthalene, early increased Tn-C levels returned to normal within 6 days of injury, while in the case of the persistent injury model, Tn-C transcript levels continued to rise at later time points, with a peribronchial pattern of distribution. BO, however, was not observed in that model despite the increased Tn-C perhaps due to less severe injury or fundamental differences in airway response to injury among different rodent species. Collectively these data support further studies to determine if Tn-C actively participates in the development of bronchiolar obliteration in DA induced BO, perhaps through interactions with the epithelium or fibroblasts.

In summary, we have demonstrated that ITI of DA replicates biological, physiological and histological features of human BO. There are currently no other rodent models that replicate these features of human BO in the setting of fully vascularized aerated lung parenchyma. Using this model, we demonstrate that BO is characterized by dysregulated epithelial repair with loss of normal airway cellular composition. In addition, we demonstrate for the first time that BO involves aberrant matrix deposition of Tn-C, localized within the airway fibrotic lesions. These results provide a novel model in which mechanisms of injury leading to BO can be further elucidated, and in which plausible targets are identified to develop new therapeutic strategies.

## References

[1] Ryu JH, Myers JL, Swensen SJ (2003). Bronchiolar Disorders.. Am J Respir Crit Care Med.

[2] Chan A, Allen R (2004). Bronchiolitis Obliterans: An Update.. Curr Opin Pulm Med.

[3] Estenne M, Maurer JR, Boehler A, Egan JJ, Frost A (2002). Bronchiolitis Obliterans Syndrome 2001: An Update Of The Diagnostic Criteria.. The Journal Of Heart And Lung Transplantation.

[4] Afessa B, Litzow MR, Tefferi A (2001). Bronchiolitis Obliterans And Other Late Onset Non-Infectious Pulmonary Complications In Hematopoietic Stem Cell Transplantation.. Bone Marrow Transplant.

[5] Kreiss K, Gomaa A, Kullman G, Fedan K, Simoes EJ (2002). Clinical Bronchiolitis Obliterans In Workers At A Microwave-Popcorn Plant.. N Engl J Med.

[6] Lockey JE, Hilbert TJ, Levin LP, Ryan PH, White KL (2009). Airway Obstruction Related To Diacetyl Exposure At Microwave Popcorn Production Facilities.. European Respiratory Journal.

[7] Akpinar-Elci M, Travis WD, Lynch DA, Kreiss K (2004). Bronchiolitis Obliterans Syndrome In Popcorn Production Plant Workers.. Eur Respir J.

[8] Kim TJ, Materna BL, Prudhomme JC, Fedan KB, Enright  PL Industry-Wide Medical Surveillance Of California Flavor Manufacturing Workers: Cross-Sectional Results.. American Journal Of Industrial Medicine.

[9] Van Rooy FGBGJ, Rooyackers JM, Prokop M, Houba R, Smit LAM (2007). Bronchiolitis Obliterans Syndrome In Chemical Workers Producing Diacetyl For Food Flavorings.. Am J Respir Crit Care Med.

[10] Hubbs AF, Goldsmith WT, Kashon ML, Frazer D, Mercer RR (2008). Respiratory Toxicologic Pathology Of Inhaled Diacetyl In Sprague-Dawley Rats.. Toxicologic Pathology.

[11] Morgan DL, Flake GP, Kirby PJ, Palmer SM (2008). Respiratory Toxicity Of Diacetyl In C57Bl/6 Mice.. Toxicol Sci.

[12] Morris JB, Hubbs AF (2009). Inhalation Dosimetry Of Diacetyl And Butyric Acid, Two Components Of Butter Flavoring Vapors.. Toxicological Sciences.

[13] Agostini C, Gurrieri C (2006). Chemokine/Cytokine Cocktail In Idiopathic Pulmonary Fibrosis.. Proc Am Thorac Soc.

[14] Antonov J, Goldstein DR, Oberli A, Baltzer A, Pirotta M (2005). Reliable Gene Expression Measurements From Degraded RNA By Quantitative Real-Time PCR Depend On Short Amplicons And A Proper Normalization.. Lab Invest.

[15] Mcdyer JF (2007). Human And Murine Obliterative Bronchiolitis In Transplant.. Proc Am Thorac Soc.

[16] Costa CL, Spilborghs GM, Martins MA, Saldiva PH, Mauad T (2005). Nitric Acid-Induced Bronchiolitis In Rats Mimics Childhood Bronchiolitis Obliterans.. Respiration.

[17] Appel JZ, Lee SM, Hartwig MG, Li B, Hsieh CC (2007). Characterization Of The Innate Immune Response To Chronic Aspiration In A Novel Rodent Model.. Respir Res.

[18] Glaab T, Taube C, Braun A, Mitzner W (2007). Invasive And Noninvasive Methods For Studying Pulmonary Function In Mice.. Respir Res.

[19] Akpinar-Elci M, Stemple KJ, Enright PL, Fahy JV, Bledsoe TA (2005). Induced Sputum Evaluation In Microwave Popcorn Production Workers.. Chest.

[20] Verleden GM, Vos R, De Vleeschauwer SI, Willems-Widyastuti A, Verleden SE (2009). Obliterative Bronchiolitis Following Lung Transplantation: From Old To New Concepts?. Transplant International.

[21] Vanaudenaerde BM, Meyts I, Vos R, Geudens N, De Wever W (2008). A Dichotomy In Bronchiolitis Obliterans Syndrome After Lung Transplantation Revealed By Azithromycin Therapy.. European Respiratory Journal.

[22] Chen H, Matsumoto K, Stripp BR (2009). Bronchiolar Progenitor Cells.. Proc Am Thorac Soc.

[23] Nord M, Schubert K, Cassel TN, Andersson O, Riise GC (2002). Decreased Serum And Bronchoalveolar Lavage Levels Of Clara Cell Secretory Protein (CC16) Is Associated With Bronchiolitis Obliterans Syndrome And Airway Neutrophilia In Lung Transplant Recipients.. Transplantation.

[24] Mattsson J, Remberger M, Andersson O, Sundberg B, Nord M (2005). Decreased Serum Levels Of Clara Cell Secretory Protein (CC16) Are Associated With Bronchiolitis Obliterans And May Permit Early Diagnosis In Patients After Allogeneic Stem-Cell Transplantation.. Transplantation.

[25] Karjalainen EM, Lindqvist A, Laitinen LA, Kava T, Altraja A (2003). Airway Inflammation And Basement Membrane Tenascin In Newly Diagnosed Atopic And Nonatopic Asthma.. Respiratory Medicine.

[26] Laitinen A, Altraja A, Kampe M, Linden M, Virtanen I (1997). Tenascin Is Increased In Airway Basement Membrane Of Asthmatics And Decreased By An Inhaled Steroid.. Am J Respir Crit Care Med.

[27] Nakahara H, Gabazza E, Fujimoto H, Nishii Y, D'Alessandro-Gabazza C (2006). Deficiency Of Tenascin C Attenuates Allergen-Induced Bronchial Asthma In The Mouse.. European Journal Of Immunology.

[28] Snyder JC, Zemke AC, Stripp BR (2009). Reparative Capacity Of Airway Epithelium Impacts Deposition And Remodeling Of Extracellular Matrix.. Am J Respir Cell Mol Biol.

